# A Latent Profile Analysis of Emotion Regulation in Relation to Distress of Symptoms in Youth with Prodromal Psychotic Symptoms

**DOI:** 10.3390/bs14080698

**Published:** 2024-08-11

**Authors:** Peiyu Zhang, Manling Long, Jingyu Shi

**Affiliations:** 1 School of Medicine, Tongji University, Shanghai 200331, China; zhangpy@tongji.edu.cn (P.Z.); 2311053@tongji.edu.cn (M.L.); 2 Clinical Research Center for Mental Disorders, Shanghai Pudong New Area Mental Health Center, School of Medicine, Tongji University, Shanghai 200124, China; 3 Department of Medical Humanities and Behavioral Sciences, School of Public Health, School of Medicine, Tongji University, Shanghai 200331, China

**Keywords:** latent profile analysis, distress of symptoms, emotion regulation, early psychological intervention

## Abstract

(1) Background: Although abnormal emotion regulation is a key characteristic of youth with prodromal psychotic symptoms and is closely related to the degree of distress caused by these symptoms, research on the internal heterogeneity of emotion regulation within this clinically high-risk population has been insufficient. (2) Methods: The current study analyzed data from 394 college students with prodromal psychotic symptoms, using latent profile analysis (LPA) to identify emotion regulation profiles based on differences in expressive suppression and cognitive reappraisal. One-way ANOVA was employed to examine the relationship between the identified latent profiles and distress of symptoms. (3) Results: Three latent profiles were identified: “high cognitive reappraisal group” (*n* = 117, 29.70%), “moderate cognitive reappraisal group” (*n* = 233, 59.14%), and “low cognitive reappraisal group” (*n* = 44, 11.16%). Significant differences in distress of negative symptoms (*F* = 9.122, *p* < 0.05) and perceptual abnormalities (*F* = 3.103, *p* < 0.05) were observed across the latent profiles but not in unusual thought content and specific perceptual abnormalities (both *p* > 0.05). The “low cognitive reappraisal group” exhibited the most severe distress of symptoms, followed by the “moderate cognitive reappraisal group”, while the “high cognitive reappraisal group” experienced the least distress. (4) Conclusions: The current study provides evidence for the heterogeneity of emotion regulation among youth with prodromal psychotic symptoms and reveals differences in distress of perceptual abnormalities and negative symptoms between the latent profiles of emotion regulation. These findings support the development of targeted psychological interventions to alleviate the distress of symptoms and improve quality of life.

## 1. Introduction

Prodromal psychotic symptoms, which include abnormalities in perception, thinking, behavior, and emotions, typically occur during adolescence or early adulthood and are early indicators of schizophrenia [1]. It is worth noting that the occurrence of such attenuated psychotic symptoms does not necessarily mean that an individual will inevitably develop schizophrenia in the future [2]. However, experiencing these symptoms can negatively impact the psychological health and social functioning of youths [3,4]. These symptoms are usually associated with impaired functioning and varying degrees of psychological distress [5,6]. From a biopsychosocial perspective, interventions for youth with prodromal psychotic symptoms should prioritize not only decreasing the prodromal symptoms but also reducing distress of symptoms to improve quality of life [7,8,9]. While a growing number of researchers recognize the importance of reducing distress of symptoms in early interventions, there is still a noticeable lack of research on the mechanisms underlying the emergence of distress of symptoms, which hinders the development of effective interventions [10,11]. Thus, it is crucial to investigate the distress of symptoms experienced by youths with prodromal psychotic symptoms and identify the associated psychological characteristics.

As youths with prodromal psychotic symptoms are in the early stages of their lives, understanding the suffering these symptoms cause is crucial for improving psychotherapeutic strategies and promoting a good prognosis [12]. The concept of distress of symptoms focuses on the psychological pain and distress caused by different prodromal psychotic symptoms [13,14]. It reflects the individual’s distressing perceptions of reality distortion and emotional discomfort, which can seriously impair sensory, cognitive, and social functioning, as well as quality of life. This includes impaired cognitive abilities, decreased daily functioning, emotional distress, and social withdrawal [15,16]. In this study, the research instrument for prodromal psychotic symptoms was the 16-item version of the Prodromal Questionnaire (PQ-16). The PQ-16 assesses four categories of prodromal psychotic symptoms: perceptual abnormalities, thought content abnormalities, specific perceptual abnormalities, and negative symptoms [17]. Furthermore, it assesses the degree of symptom distress. Consequently, the distress of prodromal psychotic symptoms in the present study was classified according to the categories examined by the PQ-16: distress of perceptual abnormalities, unusual thought content, specific perceptual abnormalities, and negative symptoms. Although relative studies have revealed the adverse conditions faced by youths with prodromal psychosis and their relevance to distress of symptoms, the mechanisms of prodromal distress remain poorly understood.

Improving abnormal emotional experiences and establishing adjusted emotion regulation strategies are considered as important tools for early intervention in youths with prodromal psychotic symptoms [18]. Emotion regulation refers to the ability of a person to recognize, evaluate, and adapt emotional experiences to internal and external circumstances. According to the process model of emotion regulation, cognitive reappraisal and expressive suppression are the two most widely used and representative emotion regulation strategies [19]. Expressive suppression refers to an emotion regulation strategy where individuals deliberately inhibit or hide the external expression of emotional reactions. While expressive suppression can help reduce interpersonal conflicts and disputes, it prevents effective emotional expression and processing. This can lead to the accumulation of unprocessed emotions, resulting in psychological distress such as anxiety and depression. Consequently, expressive suppression is often considered a maladaptive emotion regulation strategy [20]. Cognitive reappraisal involves an individual’s reinterpretation of a situation or emotional experience to alter their subjective emotional response, which, through re-evaluating and reinterpreting situations or events, can reduce negative emotional reactions or enhance positive emotional reactions. This helps individuals better manage their emotions and enhances their psychological resilience. Therefore, it is considered a positive and effective emotion regulation strategy [21]. Studies have demonstrated that individuals with prodromal psychotic symptoms show significant deficits in emotion regulation, such as having high emotional reactivity and difficulties in the rational use of emotion regulation strategies [22,23]. Individuals in the prodromal stage of psychosis tend to employ more suppression strategies like avoidance, denial, and delusion to cope with stress while showing a reduced inclination towards cognitive reappraisal. Some studies also reveal the correlation between expressive suppression and the distress of symptoms [24,25,26].

Although these studies have been conducted to reveal common features in emotion regulation in groups with prodromal psychotic symptoms, the potential heterogeneity of emotion regulation within the prodromal population has been less explored. Latent profile analysis (LPA), as a person-centered research method, can identify latent, non-overlapping subgroups within data [27]. By classifying individuals with similar emotion regulation characteristics into the same latent profile based on their response patterns to two dimensions of emotion regulation (expression suppression and cognitive reappraisal), LPA helps to uncover the internal heterogeneity of emotion regulation in youth with prodromal psychotic symptoms. Furthermore, it allows for the exploration of differences in distress of symptoms among different emotion regulation subgroups. This facilitates the development of tailored psychological interventions for each subgroup, thereby enhancing the efficiency of targeted interventions.

The aim of the current study was to identify different latent profiles of emotion regulation in youth with prodromal psychotic symptoms and to examine the relationship between these potential profiles and distress of symptoms. Ultimately, it provides a reference by which to promote individualized psychological interventions for reducing distress and improving quality of life.

## 2. Materials and Methods

### 2.1. Participants and Procedure

A cross-sectional survey was conducted at a comprehensive university in Shanghai, China, utilizing a whole-cluster random sampling method to generate a sample of all college freshmen. A total of 6357 individuals participated in the survey. After excluding invalid questionnaires and those from individuals with a diagnosed mental illness, 6314 questionnaires were included in the analysis. Of these, 394 individuals (6.24%), with a PQ-16 item score of 9 or more, were identified as having prodromal psychotic symptoms according to a previous study by our team [28].

Ethical approval was obtained from the Ethics Committee of the Institutional Review Board of Tongji University (No. 2016yxy07). All procedures were performed in accordance with the Declaration of Helsinki, and written informed consent was obtained from all participants.

### 2.2. Measures

#### 2.2.1. Sociodemographic Questionnaire

The self-designed questionnaire which was developed for this study was used to obtain sociodemographic variables, including age, gender, left behind experience (children being left behind in their hometowns by parents who have migrated for work purposes), only child or not, and anamnesis of mental disease.

#### 2.2.2. The 16-Item Version of Prodromal Questionnaire (PQ-16)

The Chinese version of the PQ-16 [29] was used to assess prodromal psychotic symptoms. This 16-item self-report scale includes four dimensions: perceptual abnormalities, unusual thought content, specific perceptual abnormalities, and negative symptoms (the first three dimensions represent positive symptoms). The PQ-16 scores were divided into item scores and distress scores. Item scores determined whether participants experienced prodromal psychotic symptoms, while distress scores measured the degree of distress caused by these symptoms.

Participants reported their psychotic symptoms experienced within the last month, with each item marked as either yes or no. If an item was marked “no”, it was rated 0. If marked “yes”, it was rated 1, and the severity of the distress caused by the psychotic symptoms was rated on a 4-point scale (from 0 = no distress to 3 = severe distress). The PQ-16 item score was calculated as the number of items marked “yes”. The psychiatric symptom distress score was calculated based on the distress ratings for each item.

The reliability and validity of the Chinese version of the PQ-16 among Chinese college students were satisfactory [30]. In the current study, the Cronbach’s α for the PQ-16 was 0.84.

#### 2.2.3. Emotion Regulation Questionnaire (ERQ)

The Chinese version of the Emotion Regulation Questionnaire (ERQ) [31] was used to assess emotion regulation strategies. This 10-item self-report scale includes two dimensions: expression suppression and cognitive reappraisal. Each item is rated on a 7-point scale (from 1 = strongly disagree to 7 = strongly agree). Scores were calculated for each dimension, with higher scores indicating a higher frequency of using the respective emotion regulation strategy. And the total score for emotion regulation is a composite score of the two dimensional scores. The reliability and validity of the Chinese version of the ERQ among Chinese college students were satisfactory [32]. In the current study, the Cronbach’s α for the ERQ was 0.70.

### 2.3. Data Analysis

The data were analyzed using IBM SPSS 22.0 and Mplus 8.3. To mitigate potential common method bias from self-report scales, Harman’s single-factor test was conducted prior to data analysis. The test results showed that six factors had eigenvalues greater than 1, with the first factor accounting for 19.31% of the variance, which is significantly below the 40% threshold [33]. Thus, no significant common method bias was detected in this study.

Data analysis consisted of three parts. First, descriptive statistics of the sample’s sociodemographic characteristics were conducted by SPSS 22.0.

Second, a series of latent profile analyses (LPA) was conducted using Mplus 8.3 to identify distinct profiles based on participants’ responses to the two dimensions of emotion regulation: expression suppression and cognitive reappraisal. Several fit indices were used to select the best model: the Akaike information criterion (AIC), Bayesian information criterion (BIC), and adjusted Bayesian information criterion (aBIC), with lower values indicating a better fit [34]. The Lo–Mendell–Rubin likelihood ratio test (LMRT) and bootstrap likelihood ratio test (BLRT) were also used, with significant *p*-values indicating a better fit for the k-class model over the k-1 class model [35]. Entropy was calculated to assess classification accuracy, with values ideally above 0.80 [36].

Finally, the association between profile membership and distress of prodromal psychotic symptoms was assessed using one-way ANOVA in SPSS 22.0 [37].

## 3. Results

### 3.1. Participant Characteristics

In a valid sample of 394 participants, there were 241 males (61.17%) and 153 females (38.83%). The average age of the sample was 19.32 ± 2.40 years. A total of 165 participants (41.88%) had left-behind experience, and 253 participants (64.21%) were the only child in their family (Table 1).

### 3.2. Latent Profile Analysis

To explore the latent profiles of emotion regulation strategies among youth with prodromal psychotic symptoms, a latent profile analysis (LPA) was conducted based on subjects’ scores on the 10-item ERQ. Table 2 presents the model fit indices for the one-class to four-class solutions.

The AIC, BIC, and aBIC values generally decreased as the number of estimated profiles increased from one to four, while the entropy remained consistently above 0.80. This indicates that the model fit improved with the addition of more profiles. According to the LMRT and BLRT, the four-class solution did not significantly enhance model fit compared to the three-class solution (*p* > 0.05). This suggests that a solution with more than three classes does not provide a significantly better fit than the three-class solution.

Based on model fit indices and the theoretical hypothesis, the three-class solution was identified as the best representation of latent emotion regulation profiles.

As shown in Table 3, the latent profile memberships exhibited significant differences in the means of emotion regulation (*F* = 95.12, *p* < 0.01) and cognitive reappraisal (*F* = 673.72, *p* < 0.01). However, the differences in the means of expressive suppression were not statistically significant (*F* = 0.18, *p* > 0.05). Further LSD tests indicated that the differences in total scores of emotion regulation and cognitive reappraisal scores among the three groups followed a similar trend: Class 3 (*n* = 117, 29.70%) scored the highest, followed by Class 2 (*n* = 233, 59.14%), and Class 1 (*n* = 44, 11.16%) scored the lowest. This indicates that among youth with prodromal psychotic symptoms, there is no significant heterogeneity in expressive suppression scores, whereas there is significant heterogeneity in emotion regulation total scores and cognitive reappraisal scores.

**Figure 1:**
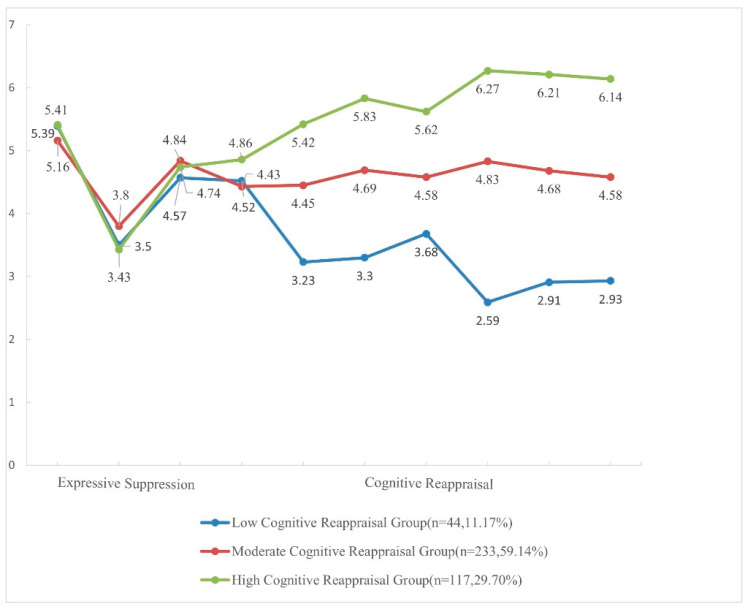
Latent profile indicators mean values for the profile solutions.

Therefore, the naming of the latent emotion regulation profiles in this study was based on the characteristic differences in cognitive reappraisal among the three latent profiles: Class 1, the “low cognitive reappraisal group”, had the lowest cognitive reappraisal scores; Class 2, the “moderate cognitive reappraisal group”, had a moderate level of cognitive reappraisal; Class 3, the “high cognitive reappraisal group”, had the highest level of cognitive reappraisal. Their characteristics are summarized in Figure 1.

### 3.3. Distress of Psychotic Symptoms across the Identified Latent Profiles

**Figure 2:**
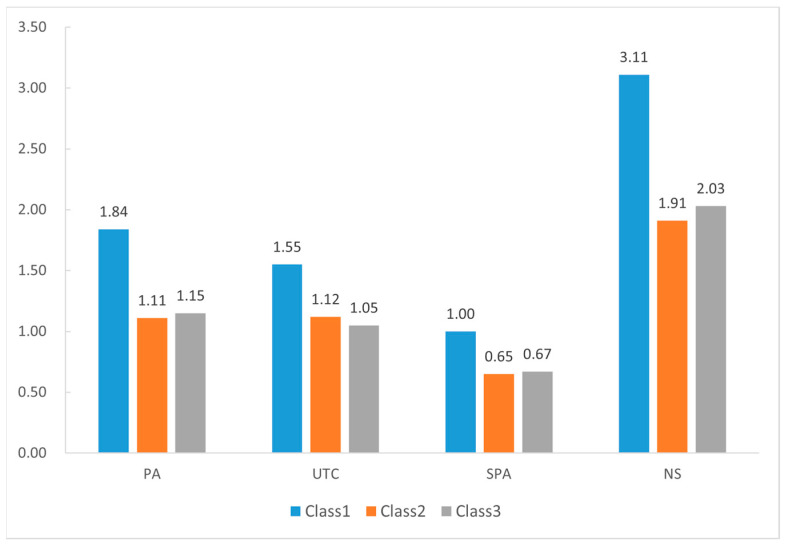
Scores on distress of perceptual abnormalities, unusual thought content, specific perceptual abnormalities, and negative symptoms for different latent profiles. Class 1 = low cognitive reappraisal group; Class 2 = moderate cognitive reappraisal group; Class 3 = high cognitive reappraisal group. PA: perceptual abnormalities; UTC: unusual thought content; SPA: specific perceptual abnormalities; NS: negative symptoms.

The mean scores of the three latent profiles on distress of perceptual abnormalities, unusual thought content, specific perceptual abnormalities, and negative symptoms are presented in Figure 2.

Furthermore, one-way ANOVA revealed significant differences between the three profiles and the distress of perceptual abnormalities (*F* = 3.103, *p* < 0.05) and negative symptoms (*F* = 6.431, *p* < 0.05). LSD test results indicated that for both perceptual abnormalities and negative symptoms scores, the “low cognitive reappraisal group” scored significantly higher than both the “moderate cognitive reappraisal group” (*p* < 0.05) and the “high cognitive reappraisal group” (*p* < 0.05). However, the differences between the moderate and high cognitive reappraisal groups were not statistically significant (*p* > 0.05).

But no significant relationship was identified between latent profiles with distress of unusual thought content (*F* = 1.765, *p* > 0.05) and specific perceptual abnormalities (*F* = 1.833, *p* > 0.05) in the *F* test.

## 4. Discussion

With the Latent profile analysis, there were three profiles identified according to the reacting patterns of emotion regulation among youth with prodromal psychotic symptoms: “high cognitive reappraisal group” (29.76%), “moderate cognitive reappraisal group” (59.14%), and “low cognitive reappraisal group” (11.17%). The results revealed that all three profiles had similar expression suppression scores, with no statistically significant differences between the scores. Youth experiencing prodromal psychotic symptoms use a similar amount of expressive suppression [38,39]. However, there is a clear difference in how much distress they feel based on the amount of cognitive reappraisal they use. The more they use cognitive reappraisal as an emotion regulation skill, the less distress they experience from psychotic symptoms. In summary, similar levels of expression suppression strategy use appear to be a common feature of emotion regulation in youth with prodromal psychotic symptoms. Additionally, different subtypes of emotion regulation can be distinguished by varying levels of cognitive reappraisal.

The present study shows that the distress caused by perceptual abnormalities and negative symptoms varies significantly across the three latent profiles. Perceptual abnormalities, such as hallucinations and illusions, are closely related to an individual’s emotional state [40]. Similarly, negative symptoms, including affective flattening, avolition, and social withdrawal, are often associated with deficits in emotion regulation abilities [38,41]. This relationship may explain the significant differences in distress from perceptual abnormalities and negative symptoms among individuals in different latent emotion regulation profiles characterized by variations in cognitive reappraisal. Moreover, specific perceptual abnormalities, such as certain types of hallucinations or perceptual distortions, are likely influenced more by specific biological and environmental factors rather than emotion regulation strategies [42]. Likewise, unusual thought content, such as delusions and bizarre thinking, typically exhibit a high degree of personalization and complexity, which may not be directly influenced by emotion regulation strategies [43]. Instead, their occurrence and persistence might involve more complex cognitive processes. Therefore, the distress caused by specific perceptual abnormalities and unusual thought content does not show significant differences among individuals in different latent emotion regulation profiles. Further studies should explore the mechanisms underlying different psychotic symptoms to confirm these results.

The current research also shows that distress of perceptual abnormalities and negative symptom were highest in the “low cognitive reappraisal group”, followed by the “moderate cognitive reappraisal group”, and lowest in the “high cognitive reappraisal group”. This further illustrates that different levels of cognitive reappraisal strategy use are associated with distress from perceptual abnormalities and negative symptoms in youth with prodromal psychotic symptoms. Cognitive reappraisal is an adaptive strategy for regulating emotions that can enhance their ability to face challenges more flexibly [21,44]. When psychotic symptoms are present, the ability of the “low cognitive reappraisal group” to adopt cognitive reappraisal as an adaptive emotion regulation strategy is hindered. This may result in their inability to effectively change their assessment of the situation or emotion in the present moment, leading to difficulty in adapting to and making sense of the symptom experience. As a result, symptoms of distress may be exacerbated [45]. In contrast, the group undergoing moderate cognitive reappraisal still demonstrated some adaptive emotion regulation skills. They were able to reduce negative emotions in the present moment and reduce symptom distress by reinterpreting the emotional experience and adjust their cognitive and affective responses [46]. The group with high cognitive reappraisal skills may be better at using effective emotion regulation strategies to adjust their perceptions of the situation. This can help to alleviate stress and negative emotions and reduce symptom distress [47]. Therefore, future research and clinical practice could focus on improving cognitive reappraisal abilities as a target for psychotherapy. By enhancing individuals’ capacity to reinterpret and rationally evaluate negative events and psychotic symptoms, it may be possible to reduce distress of perceptual abnormalities and negative symptoms, thereby improving the mental health and quality of life of youth with prodromal psychotic symptoms. For example, mindfulness training enhances individuals’ awareness of their emotions and thoughts. It helps them focus on positive emotional components and reduce attention to negative emotional triggers, thereby achieving the goal of improving emotional regulation functions [48].

## 5. Conclusions

According to the difference between reacting patterns of cognitive reappraisal, there were three latent profiles of emotion regulation identified among youth with prodromal psychotic symptoms: “high cognitive reappraisal group”, “moderate cognitive reappraisal group”, and “low cognitive reappraisal group”. Statistically significant differences in distress of perceptual abnormalities and negative symptoms were found between youth with prodromal psychotic symptoms with different latent profiles of emotion regulation. The severity of perceptual abnormalities and negative symptom distress was highest in the “low cognitive reappraisal group”, lower in the “moderate cognitive reappraisal group”, and lowest in the “high cognitive reappraisal group”. Early intervention in the prodromal stage of psychosis should focus on the individual’s emotion regulation and provide personalized interventions for youth with different types of emotion regulation. Specifically, in reducing perceptual abnormalities and negative symptoms, psychosocial interventions focused on enhancing cognitive reappraisal abilities may be an effective approach. This might help them establish better emotion regulation, reduce symptom distress, and promote the recovery of social functioning.

## 6. Limitations and Implications

This study explored differences in emotion regulation subtypes among youths in the prodromal phase of psychosis and revealed the relationship of subtypes to perceptual abnormalities and negative symptom distress, which provided a theoretical foundation for developing intervention strategies that focus on improving emotional dysfunction during the prodromal phase of psychosis. However, there are also limitations in this study. For example, this study was cross-sectional, making it difficult to explore the causal relationship between emotion regulation and individual symptom distress. Follow-up studies could use longitudinal surveys or experimental studies to reveal this relationship. Moreover, this study included only youth with prodromal psychotic symptoms and did not match a healthy control group. As a result, it is difficult to compare the latent profiles of emotion regulation between high-risk and healthy populations. Future research should consider examining the differences in the internal heterogeneity of emotion regulation between healthy individuals and those at ultra-high risk for psychosis.

## Figures and Tables

**Table 1:** Demographic of youth with prodromal psychotic symptoms (*n* = 394).

Variables	*n*	%
Age (M ± SD)	19.32 ± 2.40	
Gender		
Male	241	61.17
Female	153	38.83
Left-behind experience		
Yes	165	41.88
No	229	58.12
Only child in their family		
Yes	253	64.21
No	141	35.79

**Table 2:** Fit statistics for the latent profile analysis.

	AIC	BIC	aBIC	Entropy	LMRT *p*-Value	BLRT *p*-Value
One-class	14,086.42	14,165.94	14,102.48	-	-	-
Two-class	13,702.21	13,825.48	13,727.11	0.83	<0.05	<0.05
Three-class	13,524.91	13,691.92	13,558.65	0.82	<0.05	<0.05
Four-class	13,438.12	13,648.86	13,480.69	0.81	0.58	0.59

AIC: Akaike information criterion; BIC: Bayesian information criterion; LMRT: Lo–Mendell–Rubin likelihood ratio test; aBIC: adjusted Bayesian information criterion; BLRT: bootstrap likelihood ratio test.

**Table 3:** Descriptive statistics for emotion regulation that constituted the latent profiles.

Variables	Class 1 *M* (*SD*)	Class 2 *M* (*SD*)	Class 3 *M* (*SD*)	*F*	*LSD*
Emotion regulation	7.60 (1.69)	9.19 (1.13)	10.52 (1.30)	95.12 **	1 < 2 < 3
Expressive suppression	4.49 (1.36)	4.56 (1.00)	4.61 (1.20)	0.19	
Cognitive reappraisal	3.11 (0.53)	4.64 (0.45)	5.91 (0.43)	672.32 **	1 < 2 < 3

Class 1 = low cognitive reappraisal group; Class 2 = moderate cognitive reappraisal group; Class 3 = high cognitive reappraisal group; ** *p* < 0.01.

## Data Availability

The data presented in this study are available on request from the corresponding authors.
